# Supplementary material for: Translation affects mRNA stability in a codon-dependent manner in human cells
Source: eLife. 2019 Apr 23;8:e45396. doi: 10.7554/eLife.45396 (PMC6529216; doi:10.7554/eLife.45396)
Supplement: Figure 2—source data 1. [file elife-45396-fig2-data1.docx]

**Reporter sequences and oligos used for Figure 2**

1nt-out of frame optimal

mcherry-P2A-1nt opt, Figure 2

atggtttcaaaaggagaagaagataatatggcgataattaaagaatttatgaggtttaaagttcatatggaagggtcagttaatgggcatgaatttgaaatagaaggagaaggagaagggagaccatacgaagggacacaaacagctaaacttaaagttacaaaaggaggaccacttccatttgcatgggatatactttcaccacaatttatgtacgggtcaaaagcttacgttaaacatccagctgatataccagattaccttaaactttcatttccagaagggtttaaatgggaaagggttatgaattttgaagatggaggagttgttacagttacacaagattcatcacttcaagatggagaatttatatacaaagttaaacttagagggacaaattttccatcagatgggccagttatgcaaaaaaaaacaatgggatgggaagcgtcatcagaaaggatgtacccagaagatggggcacttaaaggggaaattaaacaaaggcttaaacttaaagatggagggcattacgatgctgaagttaaaacaacatacaaagctaaaaaaccagttcaacttccaggggcatacaatgttaatattaaacttgatataacatcacataatgaagattacacaatagttgaacaatacgaaagggcagaagggaggcattcaacaggagggatggatgaactttacaaaGGAAGCGGAGCTACTAACTTCAGCCTGCTGAAGCAGGCTGGAGACGTGGAGGAGAACCCTGGACCTCTCGAGGACATCTTTGGCTTTGAGAACTTTGAGGTCAACCGCTTTGAGCAGTTCAACATTAACTATGCAAACGAGAAGCTTCAGGAGTATTTCAACAAGCACATTTTCTCACTGGAGCAGCTTGAGTTCAGGAAGGTGCAGCATGAGCTGGAGGAGGCTCAGGAGAGAGCTGACATCGCCGAGTCCCAGGTCAACAAGCTCAGAGCTAAAAGCCGTGAATTTGGAAAGGGTAAAGAGGCTGAGGAGGCTGACTCCTTCGACTATAAGAGCTTCTTCGCCAAGGTTGGGCTGTCCGCCAAGACTCCTGATGACATCAAGAAGGCTTTTGCTGTCATTGACCAGGACAAGAGCGGCTTCATTGAGGAGGATGTGGAGGACTCCCTCTGTGAGGCCAAAGAGCTGTTCATCAAGACAGTCAAGCACTTCGGTGAGGACGCTGATAAGATGCAGCCTGATGAGTTCTTTGGGATTTTCGACCAGTTCTTGCGTATCCCCAAGGAGCAGGGCTTCCTGTCGTTCTGGAGAGGAAACTTGGCCAACGTCATCAGATACTTCCCCACACAGGCCCTCAACTTTGCTTTCAAGGACAAGTACAAGAAGGTCTTCGACATCACAGACAAGCTGGAGAACGAGCTGGCCAATAAGGAGGCTTTCCTCAGACAGATGGAGGAGAAGAACAGGCAGTTGCAGGAGCGGCTTGAGTTGGCAGAGCAGAAGCTCCAGCAGTCTAGAGATTACAAAGATGACGACGATAAATGAGATCGGAAGAGCACACGTCTGAACTCCAGTCACActagataCAGGCATAGGATGACAAAGGGAAgtcgaCAGGCATAGGATGACAAAGGGAAgtcgaCAGGCATAGGATGACAAAGGGAAgtcCTAGAactatagtgagtcgtattacgtagatccagacatgataagatacattgatgagtttggacaaaccacaactagaatgcagtgaaaaaaatgctttatttgtgaaatttgtgatgctattgctttatttgtaaccattataagctgcaataaacaagtt

1. 1nt-out of frame non-optimal

mcherry-P2A-1nt non, Figure 2

atggtttcaaaaggagaagaagataatatggcgataattaaagaatttatgaggtttaaagttcatatggaagggtcagttaatgggcatgaatttgaaatagaaggagaaggagaagggagaccatacgaagggacacaaacagctaaacttaaagttacaaaaggaggaccacttccatttgcatgggatatactttcaccacaatttatgtacgggtcaaaagcttacgttaaacatccagctgatataccagattaccttaaactttcatttccagaagggtttaaatgggaaagggttatgaattttgaagatggaggagttgttacagttacacaagattcatcacttcaagatggagaatttatatacaaagttaaacttagagggacaaattttccatcagatgggccagttatgcaaaaaaaaacaatgggatgggaagcgtcatcagaaaggatgtacccagaagatggggcacttaaaggggaaattaaacaaaggcttaaacttaaagatggagggcattacgatgctgaagttaaaacaacatacaaagctaaaaaaccagttcaacttccaggggcatacaatgttaatattaaacttgatataacatcacataatgaagattacacaatagttgaacaatacgaaagggcagaagggaggcattcaacaggagggatggatgaactttacaaaGGAAGCGGAGCTACTAACTTCAGCCTGCTGAAGCAGGCTGGAGACGTGGAGGAGAACCCTGGACCTCTCGAGACATCTTTGGCTTTGAGAACTTTGAGGTCAACCGCTTTGAGCAGTTCAACATTAACTATGCAAACGAGAAGCTTCAGGAGTATTTCAACAAGCACATTTTCTCACTGGAGCAGCTTGAGTTCAGGAAGGTGCAGCATGAGCTGGAGGAGGCTCAGGAGAGAGCTGACATCGCCGAGTCCCAGGTCAACAAGCTCAGAGCTAAAAGCCGTGAATTTGGAAAGGGTAAAGAGGCTGAGGAGGCTGACTCCTTCGACTATAAGAGCTTCTTCGCCAAGGTTGGGCTGTCCGCCAAGACTCCTGATGACATCAAGAAGGCTTTTGCTGTCATTGACCAGGACAAGAGCGGCTTCATTGAGGAGGATGTGGAGGACTCCCTCTGTGAGGCCAAAGAGCTGTTCATCAAGACAGTCAAGCACTTCGGTGAGGACGCTGATAAGATGCAGCCTGATGAGTTCTTTGGGATTTTCGACCAGTTCTTGCGTATCCCCAAGGAGCAGGGCTTCCTGTCGTTCTGGAGAGGAAACTTGGCCAACGTCATCAGATACTTCCCCACACAGGCCCTCAACTTTGCTTTCAAGGACAAGTACAAGAAGGTCTTCGACATCACAGACAAGCTGGAGAACGAGCTGGCCAATAAGGAGGCTTTCCTCAGACAGATGGAGGAGAAGAACAGGCAGTTGCAGGAGCGGCTTGAGTTGGCAGAGCAGAAGCTCCAGCAGGTCTAGAGATTACAAAGATGACGACGATAAATGAGATCGGAAGAGCACACGTCTGAACTCCAGTCACActagataCAGGCATAGGATGACAAAGGGAAgtcgaCAGGCATAGGATGACAAAGGGAAgtcgaCAGGCATAGGATGACAAAGGGAAgtcCTAGAactatagtgagtcgtattacgtagatccagacatgataagatacattgatgagtttggacaaaccacaactagaatgcagtgaaaaaaatgctttatttgtgaaatttgtgatgctattgctttatttgtaaccattataagctgcaataaacaagtt

1. Extreme optimal

cardinal-P2A-ex opt, Figure 2

atggtttcaaaaggagaagaacttattaaagaaaatatgcatatgaaactttatatggaaggaacagttaataatcatcatttcaaatgtacaacagaaggagaaggaaaaccatatgaaggaacacaaacacaaagaattaaagttgttgaaggaggaccacttccattcgcattcgatattcttgcaacatgtttcatgtatggatcaaaaacattcattaatcatacacaaggaattccagatttcttcaaacaatcattcccagaaggattcacatgggaaagagttacaacatatgaagatggaggagttcttacagttacacaagatacatcacttcaagatggatgtcttatttataatgttaaacttagaggagttaatttcccatcaaatggaccagttatgcaaaaaaaaacacttggatgggaagcgacaacagaaacactttatccagcagatggaggacttgaaggaagatgtgatatggcacttaaacttgttggaggaggacatcttcattgtaatcttaaaacaacatacagatcaaaaaaaccagcgaaaaatcttaaaatgccaggagtttacttcgttgatagaagacttgaaagaattaaagaagcagataatgaaacatacgttgaacaacatgaagttgcagttgcaagatactgtgatcttccatcaaaacttggacataaacttaatggaatggatgaactttacaaaGGAAGCGGAGCTACTAACTTCAGCCTGCTGAAGCAGGCTGGAGACGTGGAGGAGAACCCTGGACCTCTCGAGatggccggcgataacggacccgaagatcgtgacaacggcgacgatggaggttatgctggaaagggagtcggaggcccaaaccctggaaacggcaccttccctggggggttctacggttattatggagccaagggggatttcgacatcgtcgctttcgggtactatggccgtcctatcggacctgggatcattcagaacttcgatgctgcttacgccgctgctatgccaattgagaaggaagatcccgctccatatattttccaggggggtaacgaaaagaacggaaccgctatcgtcggcgatgcaggaatggaaaaggatgactatggggaggaggtcgatcccgacccaatcatggatatgaacggtgagaccggggcatacaaggctgccgacgccggtacccgttatggtgaaatggaacccgctgccgaagatttcgccgacgaccaggagccaccagcctatgtcttcatcattaaggacatgcagggtccctattatgcagccaacttcggggaggacggtttcgaaggagctaaggatttcggcatgaccaacaccggcggtggtaaccgtgagatgaaggggtatgagttcgaacaggccgaagacggggaaaagcgtgaagaggaggagcctggcgacattaagtacatgggctatggtaacgccaaggctgccggaggccagattgagatggcaatgggcggtgcagggTCTAGAGATTACAAAGATGACGACGATAAATGAGATCGGAAGAGCACACGTCTGAACTCCAGTCACActagataCAGGCATAGGATGACAAAGGGAAgtcgaCAGGCATAGGATGACAAAGGGAAgtcgaCAGGCATAGGATGACAAAGGGAAgtcCTAGAactatagtgagtcgtattacgtagatccagacatgataagatacattgatgagtttggacaaaccacaactagaatgcagtgaaaaaaatgctttatttgtgaaatttgtgatgctattgctttatttgtaaccattataagctgcaataaacaagtt

1. extreme non-optimal

cardinal-P2A-ex non, Figure 2

atggtttcaaaaggagaagaacttattaaagaaaatatgcatatgaaactttatatggaaggaacagttaataatcatcatttcaaatgtacaacagaaggagaaggaaaaccatatgaaggaacacaaacacaaagaattaaagttgttgaaggaggaccacttccattcgcattcgatattcttgcaacatgtttcatgtatggatcaaaaacattcattaatcatacacaaggaattccagatttcttcaaacaatcattcccagaaggattcacatgggaaagagttacaacatatgaagatggaggagttcttacagttacacaagatacatcacttcaagatggatgtcttatttataatgttaaacttagaggagttaatttcccatcaaatggaccagttatgcaaaaaaaaacacttggatgggaagcgacaacagaaacactttatccagcagatggaggacttgaaggaagatgtgatatggcacttaaacttgttggaggaggacatcttcattgtaatcttaaaacaacatacagatcaaaaaaaccagcgaaaaatcttaaaatgccaggagtttacttcgttgatagaagacttgaaagaattaaagaagcagataatgaaacatacgttgaacaacatgaagttgcagttgcaagatactgtgatcttccatcaaaacttggacataaacttaatggaatggatgaactttacaaaGGAAGCGGAGCTACTAACTTCAGCCTGCTGAAGCAGGCTGGAGACGTGGAGGAGAACCCTGGACCTCTCGAGcgaagtcaatcactgtcgacgtctctatcgctaaggcgcgtacgaagtttgaaacggagggcgcttctcccgctcagacgcaaaacagtatgcttttgtcatatattgaggcatccgacgagagtggttctttgcctcacaactgcgagatttcgatgtagcatatctgttgcgataaatagtaattgcgtatctttacatctgcaccataattcgtgggtttggttaaggcatagaagctcacacctgtgtcggccgagccatgtacaatctttacttcgctgtaaaacttctaggtcgaggctcctgcaaacgtccacgttatcatgctgtaattcacgatcgagactatgttcccatcttgtgtccaaatgcttaacactacaacgcaggctaataatactattgcaacttgtgactttgactagctcagtggttcggcacttgaattggacagttcaccaaactacgagtcatacatggctcactaataaatggacgttgcactctacaccgagctttactcgctcacgaccgacggcgttatccctttttacgagttccatacttgtatggtcttcccacctgtgcctcagactgctacgacaatttacacactcccgaagcgtgctacaccggctcaggatatgcgcgctgtcacggtggcgcgtaaaaatacgacaaagcagtccgttgagttgtagcacattagtaactagtTCTAGAGATTACAAAGATGACGACGATAAATGAGATCGGAAGAGCACACGTCTGAACTCCAGTCACActagataCAGGCATAGGATGACAAAGGGAAgtcgaCAGGCATAGGATGACAAAGGGAAgtcgaCAGGCATAGGATGACAAAGGGAAgtcCTAGAactatagtgagtcgtattacgtagatccagacatgataagatacattgatgagtttggacaaaccacaactagaatgcagtgaaaaaaatgctttatttgtgaaatttgtgatgctattgctttatttgtaaccattataagctgcaataaacaagtt

1. another 1nt- out of frame opt/non.

New 1nt- out of frame optimal Figure 2-figure supplement 1

ACTTCCTTTGGAAGCTTCAGGCCACAGAAGAGAACGAGGAGATGGAGGAACTTCAAGCCTACAACCGCCGGCTACTCCACAACATCCTGCCCAAAGACGTGGCTGCGCACTTTCTGGCCCCAGACCAGCCTGTTCTTTGACAGGTTTGTGCAGTGGAAGAAGTTTGAAAAGCAGCCCATCACTGATAAATACTTCTATGAGTTCAGGACATTGGGGAAAGGAGGATTTGGAGAGGTATGTGATGTCATTTTCTACATGGCTGCTGTGTTTTGGACCTTCACTGTCCTGTATAAAGGCCACATTTCCTTGGGAGAATCCGTGGGTTATTTGGGGATGTATATTGCATATGTTTTTACTGTGCGATTCGCCTATGTCCGCGACTTCTGGACCACCTTCGTGGAGACCCGATGGCGGTTCATACTCTTGTATTTTGTGGCATCCTTTACCCTCAGCTGGTTTATCTTCGGACTCATCTGGTACAGGAGGACAGGGTATTATACAGTGTATAAACAGGCATACAACATGGAGATGCAGACCGTCTTCAGGTGCTGCCCAGGATGGATGCAGAGAGGAGAAGAGAGAGGCTGTTTGCACAGAGTGTTCATCTCCAGACCCATCGCAGTGCTCAACATCATACTCAACATGGTGCTGTACACGATGCAGGGCGGTAACTTTAAACAGGCTTTTGTGGAGCTCTTCAAATGTAAGAAACTCAAGTCG

New 1nt- out of frame non-optimal, Figure 2-figure supplement 1

CTTCCTTTGGAAGCTTCAGGCCACAGAAGAGAACGAGGAGATGGAGGAACTTCAAGCCTACAACCGCCGGCTACTCCACAACATCCTGCCCAAAGACGTGGCTGCGCACTTTCTGGCCCCAGACCAGCCTGTTCTTTGACAGGTTTGTGCAGTGGAAGAAGTTTGAAAAGCAGCCCATCACTGATAAATACTTCTATGAGTTCAGGACATTGGGGAAAGGAGGATTTGGAGAGGTATGTGATGTCATTTTCTACATGGCTGCTGTGTTTTGGACCTTCACTGTCCTGTATAAAGGCCACATTTCCTTGGGAGAATCCGTGGGTTATTTGGGGATGTATATTGCATATGTTTTTACTGTGCGATTCGCCTATGTCCGCGACTTCTGGACCACCTTCGTGGAGACCCGATGGCGGTTCATACTCTTGTATTTTGTGGCATCCTTTACCCTCAGCTGGTTTATCTTCGGACTCATCTGGTACAGGAGGACAGGGTATTATACAGTGTATAAACAGGCATACAACATGGAGATGCAGACCGTCTTCAGGTGCTGCCCAGGATGGATGCAGAGAGGAGAAGAGAGAGGCTGTTTGCACAGAGTGTTCATCTCCAGACCCATCGCAGTGCTCAACATCATACTCAACATGGTGCTGTACACGATGCAGGGCGGTAACTTTAAACAGGCTTTTGTGGAGCTCTTCAAATGTAAGAAACTCAAGTCGA

Primers for qPCR for Figure 2-figure supplement 1

mchery 5’

GGATGGGAAGCGTCATCAGA

mcherry 3’

AGCATCGTAATGCCCTCCAT

GFP 5’

TGGGTCAGTTCAACTTGCAG

GFP 3’

GAAAGCGCTGATTGTGTTGA

cardinal 5’

TTCCCAGAAGGATTCACATGG

cardinal 3’

GTCCTCCATCTGCTGGATAAAG

Probes for Northern blot Figure 2

P2A anti probe

CACGTCTCCAGCCTGCTTCAGCAGGCTGAAGTTAG + 3’ biotin

299-GFP probe

gttctttcttgaacatatccttctggcattgctg+ 3’ biotin
