# Supplementary material for: Translation affects mRNA stability in a codon-dependent manner in human cells
Source: eLife. 2019 Apr 23;8:e45396. doi: 10.7554/eLife.45396 (PMC6529216; doi:10.7554/eLife.45396)
Supplement: Figure 3—source data 1. [file elife-45396-fig3-data1.docx]

**Reporter sequences and oligos used for Figure 3**

**Synonymous reporters** Figure 3BCD

Silent 100:

CGCCACCAGTCCTTCTGCGACGAGGTCTTCGTCTGCTCCGACGAGCTCAAGGTCGAACTCTCCAAGTGCCCCTGCAAGTACATCTCCCCCCACAACCCCGAACCCCAGACGCTCACGTACAACAAGCGCGTCGACAACCTCCGCATCCAGTTCCAGCGCTTCACGATCATCACGGACAACTACCACTACCACCGCGAATCCCACGACCACACGAAGGTCCTCGACCTCGTCAACTTCGAGATCGACGAAACGCAGTCCGACAACTGCAAGCAGCACTGCTACATCCAGCGCTCCTACGTCCGCATCAACGAGTCCAAGAACCCCCGCCCCACGATCTTCTTCTACTTCCCCCACTGCCTCGTCCCCCAGACGTGCTACAAGCTCCGCACGTGCTTCTGCGACGACGAGGTCCCCGAAGTCTCCAAGTGCGTCCCCTACCCCTGCATCTCCAACGACCAGCTCCACAAGCGCACGCTCTACGACCACCACTTCGAAATCCACTACCCCCAGAACGTCTACACGGAGAAGCAGAAGTCCCGCCAGAACATCTTCCGCACGCTCATCTCCTTCCTCAACCGCGACAAGACGCAGAACGTCAACCGCGAACGCTGCAACAACCACTCCCAGCCCCCCAAGGAGAAGGACGAACTCCACCCCCCCTTCATCTACATCACGTCCTGCTTCCTCAAGTACCGCCACGAGTTCGACTGCTGCTTCCAGGACATCCAGCTCATCCACGTC

Silent 75:

AGACACcagTCCTTCTGCGACGAGGTAttcGTCTGCAGTgacGAGCTCAAAGTCGAACTCTCCaagTGTCCCtgcAAGTATATCtccCCACACAACCCCGAAcccCAAACGctcACGTACAACAAGAGAgtcGACAATctcCGCATACAGttcCAGCGCTTCACAATCatcACAGATAATTACcacTATcacAGAGAATCCCACgacCATACGaagGTActcGACCTCGTCAACTTTGAGATCgacGAAACGCAAtccGACAATtgcAAGCAGCATTGCtacATACAGCGCtccTATGTCcgcATAAACGAGTCCAAGaacCCACGCCCCacgATCTTTTTCTACTTCcccCACTGTCTCgtcCCCCAAacgTGCTACAAActcAGAACGtgcTTTtgcGATGACGAGgtcCCCGAAGTAtccAAATGCgtcCCCtacCCATGCATCTCCaacGACCAGCTCCATAAGcgcACActcTACGATCACcacTTCGAAATCCACTACCCAcagAATGTCtacACGGAGAAGCAAaagAGTCGCCAGAACatcTTTCGCacgCTCATATCCTTCctcAACAGAGACaagACGCAAaacGTCAATcgcGAACGCTGTAACAACCACtccCAACCACCCAAAGAGAAGgacGAATTACACcccCCCTTTATCtacATAacgTCCTGCTTCCTCAAGTACCGCCATGAGTTCgacTGCTGTttcCAGGACATCCAGCTCATAcacGTATCTAGAG

Silent 50:

AGACACCAATCCTTCTGCGACGAGGTATTTGTCTGCAGTGATGAGCTCAAAGTCGAACTCTCCAAATGTCCCTGTAAGTATATCAGTCCACACAACCCCGAACCACAAACGTTAACGTACAACAAGAGAGTAGACAATTTACGCATACAGTTTCAGCGCTTCACAATCATAACAGATAATTACCATTATCATAGAGAATCCCACGATCATACGAAAGTATTAGACCTCGTCAACTTTGAGATCGATGAAACGCAAAGTGACAATTGTAAGCAGCATTGCTATATACAGCGCAGTTATGTCAGAATAAACGAGTCCAAGAATCCACGCCCCACAATCTTTTTCTACTTCCCACACTGTCTCGTACCCCAAACATGCTACAAATTAAGAACGTGTTTTTGTGATGACGAGGTACCCGAAGTAAGTAAATGCGTCCCCTATCCATGCATCTCCAATGACCAGCTCCATAAGAGAACATTATACGATCACCATTTCGAAATCCACTACCCACAAAATGTCTATACGGAGAAGCAAAAAAGTCGCCAGAACATATTTCGCACACTCATATCCTTCTTAAACAGAGACAAAACGCAAAATGTCAATAGAGAACGCTGTAACAACCACAGTCAACCACCCAAAGAGAAGGATGAATTACACCCACCCTTTATCTATATAACATCCTGCTTCCTCAAGTACCGCCATGAGTTCGATTGCTGTTTTCAGGACATCCAGCTCATACATGTA

Silent 25:

AGACACCAAagtTTCtgtGACgaaGTATTTGTCtgtAGTGATGAGttaAAAGTCGAACTCagtAAATGTCCCTGTaaaTATATCAGTCCAcatAACccaGAACCACAAacaTTAACGtatAACaaaAGAGTAGACAATTTAagaATACAGTTTCAGagaTTCACAataATAACAGATAATTACCATTATCATAGAGAATCCcatGATCATACGAAAGTATTAgatCTCGTCAACTTTgaaATCGATGAAACGCAAAGTgatAATTGTAAGcaaCATTGCTATATAcaaCGCAGTTATgtaAGAATAAACgaaTCCaaaAATCCACGCccaACAATCTTTtttTACtttCCACACTGTttaGTACCCCAAACAtgtTACAAATTAAGAacaTGTTTTTGTGATGACgaaGTACCCGAAGTAAGTAAATGCgtaCCCTATCCAtgtATCagtAATGACcaaCTCCATaaaAGAACATTATACGATcatCATTTCGAAataCACtatCCACAAAATGTCTATacaGAGaaaCAAAAAAGTCGCcaaAACATATTTCGCACAttaATATCCtttTTAAACAGAgatAAAACGCAAAATgtaAATAGAGAAagaTGTAACaatCACAGTCAACCAccaAAAGAGaaaGATGAATTAcatCCACCCTTTataTATATAACATCCtgtTTCttaAAGTACCGCCATgaaTTCGATtgtTGTTTTCAGgatATCcaaCTCATACATGTATCTAGAG

**Probes for Northern** Figure 3C

P2A anti probe

CACGTCTCCAGCCTGCTTCAGCAGGCTGAAGTTAG + 3’ biotin

299-GFP probe

gttctttcttgaacatatccttctggcattgctg+ 3’ biotin

**Mini-gene** GFP-P2A-mini gene, Figure 3EF

Mini-gene CTG

GGCCGGATCCTCTAACGGCGAAatgtcaaaaggagaagaacttttcacaggggttgttccaatacttgttgaacttgatggggatgttaatgggcataaattctcagtttcaggagaaggagaaggagaagcgacatatgggaaacttacacttaaattcatatgtacaacagggaaacttccagttccatggccaacacttgttacaacacttacatatggagttcaatgtttctcaagatatccagatcatatgaaacaacatgatttcttcaaatcagcaatgccagaaggatatgttcaagaaagaacaatattcttcaaagatgatgggaattataaaacaagagctgaagttaaatttgaaggggatacacttgttaatagaattgaacttaaaggaattgaatttaaagaagatgggaatatacttgggcataaacttgaatataattataattcacataatgtttatattatggcagataaacaaaaaaatgggattaaagttaattttaaaattagacataatattgaagatgggtcagttcaacttgcagatcattatcaacaaaatacaccaattggggatgggccagttcttcttccagataatcattatctttcaacacaatcagcgctttcaaaagaaccaaatgaaaaaagagatcatatggttcttcttgaatttgttacagcagcagggattacacatgggatggatgaactttacaaaGGAAGCGGAGCTACTAACTTCAGCCTGCTGAAGCAGGCTGGAGACGTGGAGGAGAACCCTGG*ACCTCTCGAGT*CTCCTGCAGGcacc**ATG**CTGGTACTGCGACTGCAACTGGCTCTGGCGCTGGTCCTGACCCTGCTACTGGCCCTGAAACTGAGGCTGAGTCTGCTTCTGATCCTGTTCCTGACGCTGGGACTGCCTCTGCGCCTGTGGCTGCGTCTGGTTCTGCAGCTGTGCCTGGAGCTGCCGCTGGGCCTGTCTCTGTTACTGTTACTGGGGCTGACTCTGCCCCTGCATCTGGTGCTGATTCTGCTCCTGAGACTGAACCTGTTTCTGTCCCTGTTGCTGGCACTGAAGCTGATACTGGACCTGCTGCTGACACTGAATCTGGATCTGAGCCTGCCACTGTACCTGTGTCTGCGGCTGTATCTGGGTCTGTCACTGGAACTGTCGCTG**TAG**AGATCGGAAGAGCACACTCTAGATACCCATACGATGTACCAGATTACGCATGAGATCGGAAGAGCACACGTCTGAACTCCAGTCAC

Mini-gene TTG

GGCCGGATCCTCTAACGGCGAAatgtcaaaaggagaagaacttttcacaggggttgttccaatacttgttgaacttgatggggatgttaatgggcataaattctcagtttcaggagaaggagaaggagaagcgacatatgggaaacttacacttaaattcatatgtacaacagggaaacttccagttccatggccaacacttgttacaacacttacatatggagttcaatgtttctcaagatatccagatcatatgaaacaacatgatttcttcaaatcagcaatgccagaaggatatgttcaagaaagaacaatattcttcaaagatgatgggaattataaaacaagagctgaagttaaatttgaaggggatacacttgttaatagaattgaacttaaaggaattgaatttaaagaagatgggaatatacttgggcataaacttgaatataattataattcacataatgtttatattatggcagataaacaaaaaaatgggattaaagttaattttaaaattagacataatattgaagatgggtcagttcaacttgcagatcattatcaacaaaatacaccaattggggatgggccagttcttcttccagataatcattatctttcaacacaatcagcgctttcaaaagaaccaaatgaaaaaagagatcatatggttcttcttgaatttgttacagcagcagggattacacatgggatggatgaactttacaaaGGAAGCGGAGCTACTAACTTCAGCCTGCTGAAGCAGGCTGGAGACGTGGAGGAGAACCCTGG*ACCTCTCGAGT*CTCCTGCAGGcacc**ATG**TTGGTATTGCGATTGCAATTGGCTTTGGCGTTGGTCTTGACCTTGCTATTGGCCTTGAAATTGAGGTTGAGTTTGCTTTTGATCTTGTTCTTGACGTTGGGATTGCCTTTGCGCTTGTGGTTGCGTTTGGTTTTGCAGTTGTGCTTGGAGTTGCCGTTGGGCTTGTCTTTGTTATTGCACTTGGGGTTGACTTTGCCCTTGCATTTGGTGTTGATTTTGCTCTTGAGATTGAACTTGTTTTTGTCCTTGTTGTTGGCATTGAAGTTGATATTGGACTTGCTGTTGACATTGAATTTGGATTTGAGCTTGCCATTGTACTTGTGTTTGCGGTTGTATTTGGGTTTGTCATTGGAATTGTCGTTG**TAG**AGATCGGAAGAGCACACTCTAGATACCCATACGATGTACCAGATTACGCATGAGATCGGAAGAGCACACGTCTGAACTCCAGTCAC

Mini-gene CTT

GGCCGGATCCTCTAACGGCGAAatgtcaaaaggagaagaacttttcacaggggttgttccaatacttgttgaacttgatggggatgttaatgggcataaattctcagtttcaggagaaggagaaggagaagcgacatatgggaaacttacacttaaattcatatgtacaacagggaaacttccagttccatggccaacacttgttacaacacttacatatggagttcaatgtttctcaagatatccagatcatatgaaacaacatgatttcttcaaatcagcaatgccagaaggatatgttcaagaaagaacaatattcttcaaagatgatgggaattataaaacaagagctgaagttaaatttgaaggggatacacttgttaatagaattgaacttaaaggaattgaatttaaagaagatgggaatatacttgggcataaacttgaatataattataattcacataatgtttatattatggcagataaacaaaaaaatgggattaaagttaattttaaaattagacataatattgaagatgggtcagttcaacttgcagatcattatcaacaaaatacaccaattggggatgggccagttcttcttccagataatcattatctttcaacacaatcagcgctttcaaaagaaccaaatgaaaaaagagatcatatggttcttcttgaatttgttacagcagcagggattacacatgggatggatgaactttacaaaGGAAGCGGAGCTACTAACTTCAGCCTGCTGAAGCAGGCTGGAGACGTGGAGGAGAACCCTGG*ACCTCTCGAGT*CTCCTGCAGGcacc**ATG**CTTGTACTTCGACTTCAACTTGCTCTTGCGCTTGTCCTTACCCTTCTACTTGCCCTTAAACTTAGGCTTAGTCTTCTTCTTATCCTTTTCCTTACGCTTGGACTTCCTCTTCGCCTTTGGCTTCGTCTTGTTCTTCAGCTTTGCCTTGAGCTTCCGCTTGGCCTTTCTCTTTTACTTCACCTTGGGCTTACTCTTCCCCTTCATCTTGTGCTTATTCTTCTCCTTAGACTTAACCTTTTTCTTTCCCTTTTGCTTGCACTTAAGCTTATACTTGACCTTCTGCTTACACTTAATCTTGATCTTAGCCTTCCACTTTACCTTTGTCTTCGGCTTTATCTTGGTCTTTCACTTGAACTTTCGCTT**TAG**AGATCGGAAGAGCACACTCTAGATACCCATACGATGTACCAGATTACGCATGAGATCGGAAGAGCACACGTCTGAACTCCAGTCAC

Mini-gene ACG

GGCCGGATCCTCTAACGGCGAAatgtcaaaaggagaagaacttttcacaggggttgttccaatacttgttgaacttgatggggatgttaatgggcataaattctcagtttcaggagaaggagaaggagaagcgacatatgggaaacttacacttaaattcatatgtacaacagggaaacttccagttccatggccaacacttgttacaacacttacatatggagttcaatgtttctcaagatatccagatcatatgaaacaacatgatttcttcaaatcagcaatgccagaaggatatgttcaagaaagaacaatattcttcaaagatgatgggaattataaaacaagagctgaagttaaatttgaaggggatacacttgttaatagaattgaacttaaaggaattgaatttaaagaagatgggaatatacttgggcataaacttgaatataattataattcacataatgtttatattatggcagataaacaaaaaaatgggattaaagttaattttaaaattagacataatattgaagatgggtcagttcaacttgcagatcattatcaacaaaatacaccaattggggatgggccagttcttcttccagataatcattatctttcaacacaatcagcgctttcaaaagaaccaaatgaaaaaagagatcatatggttcttcttgaatttgttacagcagcagggattacacatgggatggatgaactttacaaaGGAAGCGGAGCTACTAACTTCAGCCTGCTGAAGCAGGCTGGAGACGTGGAGGAGAACCCTGG*ACCTCTCGAG*TCTCCTGCAGGcacc**ATG**ACGGTAACGCGAACGCAAACGGCTACGGCGACGGTCACGACCACGCTAACGGCCACGAAAACGAGGACGAGTACGCTTACGATCACGTTCACGACGACGGGAACGCCTACGCGCACGTGGACGCGTACGGTTACGCAGACGTGCACGGAGACGCCGACGGGCACGTCTACGTTAACGCACACGGGGACGACTACGCCCACGCATACGGTGACGATTACGCTCACGAGAACGAACACGTTTACGTCCACGTTGACGGCAACGAAGACGATAACGGACACGCTGACGACAACGAATACGGATACGAGCACGCCAACGTACACGTGTACGCGGACGTATACGGGTACGTCAACGGAAACGTCGACG**TAG**AGATCGGAAGAGCACACTCTAGATACCCATACGATGTACCAGATTACGCATGAGATCGGAAGAGCACACGTCTGAACTCCAGTCAC

Mini-gene ACA

GGCCGGATCCTCTAACGGCGAAatgtcaaaaggagaagaacttttcacaggggttgttccaatacttgttgaacttgatggggatgttaatgggcataaattctcagtttcaggagaaggagaaggagaagcgacatatgggaaacttacacttaaattcatatgtacaacagggaaacttccagttccatggccaacacttgttacaacacttacatatggagttcaatgtttctcaagatatccagatcatatgaaacaacatgatttcttcaaatcagcaatgccagaaggatatgttcaagaaagaacaatattcttcaaagatgatgggaattataaaacaagagctgaagttaaatttgaaggggatacacttgttaatagaattgaacttaaaggaattgaatttaaagaagatgggaatatacttgggcataaacttgaatataattataattcacataatgtttatattatggcagataaacaaaaaaatgggattaaagttaattttaaaattagacataatattgaagatgggtcagttcaacttgcagatcattatcaacaaaatacaccaattggggatgggccagttcttcttccagataatcattatctttcaacacaatcagcgctttcaaaagaaccaaatgaaaaaagagatcatatggttcttcttgaatttgttacagcagcagggattacacatgggatggatgaactttacaaaGGAAGCGGAGCTACTAACTTCAGCCTGCTGAAGCAGGCTGGAGACGTGGAGGAGAACCCTGG*ACCTCTCGAG*TCTCCTGCAGGcacc**ATG**ACAGTAACACGAACACAAACAGCTACAGCGACAGTCACAACCACACTAACAGCCACAAAAACAAGGACAAGTACACTTACAATCACATTCACAACGACAGGAACACCTACACGCACATGGACACGTACAGTTACACAGACATGCACAGAGACACCGACAGGCACATCTACATTAACACACACAGGGACAACTACACCCACACATACAGTGACAATTACACTCACAAGAACAAACACATTTACATCCACATTGACAGCAACAAAGACAATAACAGACACACTGACAACAACAAATACAGATACAAGCACACCAACATACACATGTACACGGACATATACAGGTACATCAACAGAAACATCGACA**TAG**AGATCGGAAGAGCACACTCTAGATACCCATACGATGTACCAGATTACGCATGAGATCGGAAGAGCACACGTCTGAACTCCAGTCAC

Mini-gene TGC

GGCCGGATCCTCTAACGGCGAAatgtcaaaaggagaagaacttttcacaggggttgttccaatacttgttgaacttgatggggatgttaatgggcataaattctcagtttcaggagaaggagaaggagaagcgacatatgggaaacttacacttaaattcatatgtacaacagggaaacttccagttccatggccaacacttgttacaacacttacatatggagttcaatgtttctcaagatatccagatcatatgaaacaacatgatttcttcaaatcagcaatgccagaaggatatgttcaagaaagaacaatattcttcaaagatgatgggaattataaaacaagagctgaagttaaatttgaaggggatacacttgttaatagaattgaacttaaaggaattgaatttaaagaagatgggaatatacttgggcataaacttgaatataattataattcacataatgtttatattatggcagataaacaaaaaaatgggattaaagttaattttaaaattagacataatattgaagatgggtcagttcaacttgcagatcattatcaacaaaatacaccaattggggatgggccagttcttcttccagataatcattatctttcaacacaatcagcgctttcaaaagaaccaaatgaaaaaagagatcatatggttcttcttgaatttgttacagcagcagggattacacatgggatggatgaactttacaaaGGAAGCGGAGCTACTAACTTCAGCCTGCTGAAGCAGGCTGGAGACGTGGAGGAGAACCCTGG*ACCTCTCGAGT*CTCCTGCAGGcacc**ATG**TGCGTATGCCGATGCCAATGCGCTTGCGCGTGCGTCTGCACCTGCCTATGCGCCTGCAAATGCAGGTGCAGTTGCCTTTGCATCTGCTTCTGCACGTGCGGATGCCCTTGCCGCTGCTGGTGCCGTTGCGTTTGCCAGTGCTGCTGCGAGTGCCCGTGCGGCTGCTCTTGCTTATGCCACTGCGGGTGCACTTGCCCCTGCCATTGCGTGTGCATTTGCCTCTGCAGATGCAACTGCTTTTGCTCCTGCTTGTGCGCATGCAAGTGCATATGCGACTGCCTGTGCACATGCAATTGCGATTGCAGCTGCCCATGCTACTGCTGTTGCCGGTGCTATTGCGGTTGCTCATGCGAATGCTCGTGC**TAG**AGATCGGAAGAGCACACTCTAGATACCCATACGATGTACCAGATTACGCATGAGATCGGAAGAGCACACGTCTGAACTCCAGTCAC

Mini-gene TGT

GGCCGGATCCTCTAACGGCGAAatgtcaaaaggagaagaacttttcacaggggttgttccaatacttgttgaacttgatggggatgttaatgggcataaattctcagtttcaggagaaggagaaggagaagcgacatatgggaaacttacacttaaattcatatgtacaacagggaaacttccagttccatggccaacacttgttacaacacttacatatggagttcaatgtttctcaagatatccagatcatatgaaacaacatgatttcttcaaatcagcaatgccagaaggatatgttcaagaaagaacaatattcttcaaagatgatgggaattataaaacaagagctgaagttaaatttgaaggggatacacttgttaatagaattgaacttaaaggaattgaatttaaagaagatgggaatatacttgggcataaacttgaatataattataattcacataatgtttatattatggcagataaacaaaaaaatgggattaaagttaattttaaaattagacataatattgaagatgggtcagttcaacttgcagatcattatcaacaaaatacaccaattggggatgggccagttcttcttccagataatcattatctttcaacacaatcagcgctttcaaaagaaccaaatgaaaaaagagatcatatggttcttcttgaatttgttacagcagcagggattacacatgggatggatgaactttacaaaGGAAGCGGAGCTACTAACTTCAGCCTGCTGAAGCAGGCTGGAGACGTGGAGGAGAACCCTGG*ACCTCTCGAGT*CTCCTGCAGGcacc**ATG**TGTGTATGTCGATGTCAATGTGCTTGTGCGTGTGTCTGTACCTGTCTATGTGCCTGTAAATGTAGGTGTAGTTGTCTTTGTATCTGTTTCTGTACGTGTGGATGTCCTTGTCGCTGTTGGTGTCGTTGTGTTTGTCAGTGTTGCTGTGAGTGTCCGTGTGGCTGTTCTTGTTTATGTCACTGTGGGTGTACTTGTCCCTGTCATTGTGTGTGTATTTGTCTCTGTAGATGTAACTGTTTTTGTTCCTGTTTGTGTGCATGTAAGTGTATATGTGACTGTCTGTGTACATGTAATTGTGATTGTAGCTGTCCATGTTACTGTTGTTGTCGGTGTTATTGTGGTTGTTCATGTGAATGTTCGTGT**TAG**AGATCGGAAGAGCACACTCTAGATACCCATACGATGTACCAGATTACGCATGAGATCGGAAGAGCACACGTCTGAACTCCAGTCAC

Mini-gene CAC

GGCCGGATCCTCTAACGGCGAAatgtcaaaaggagaagaacttttcacaggggttgttccaatacttgttgaacttgatggggatgttaatgggcataaattctcagtttcaggagaaggagaaggagaagcgacatatgggaaacttacacttaaattcatatgtacaacagggaaacttccagttccatggccaacacttgttacaacacttacatatggagttcaatgtttctcaagatatccagatcatatgaaacaacatgatttcttcaaatcagcaatgccagaaggatatgttcaagaaagaacaatattcttcaaagatgatgggaattataaaacaagagctgaagttaaatttgaaggggatacacttgttaatagaattgaacttaaaggaattgaatttaaagaagatgggaatatacttgggcataaacttgaatataattataattcacataatgtttatattatggcagataaacaaaaaaatgggattaaagttaattttaaaattagacataatattgaagatgggtcagttcaacttgcagatcattatcaacaaaatacaccaattggggatgggccagttcttcttccagataatcattatctttcaacacaatcagcgctttcaaaagaaccaaatgaaaaaagagatcatatggttcttcttgaatttgttacagcagcagggattacacatgggatggatgaactttacaaaGGAAGCGGAGCTACTAACTTCAGCCTGCTGAAGCAGGCTGGAGACGTGGAGGAGAACCCTGG*ACCTCTCGAGT*CTCCTGCAGGcacc**ATG**CACGTACACCGACACCAACACGCTCACGCGCACGTCCACACCCACCTACACGCCCACAAACACAGGCACAGTCACCTTCACATCCACTTCCACACGCACGGACACCCTCACCGCCACTGGCACCGTCACGTTCACCAGCACTGCCACGAGCACCCGCACGGCCACTCTCACTTACACCACCACGGGCACACTCACCCCCACCATCACGTGCACATTCACCTCCACAGACACAACCACTTTCACTCCCACTTGCACGCACACAAGCACATACACGACCACCTGCACACACACAATCACGATCACAGCCACCCACACTACCACTGTCACCGGCACTATCACGGTCACTCACACGAACACTCGCAC**TAG**AGATCGGAAGAGCACACTCTAGATACCCATACGATGTACCAGATTACGCATGAGATCGGAAGAGCACACGTCTGAACTCCAGTCAC

Mini-gene CAT

GGCCGGATCCTCTAACGGCGAAatgtcaaaaggagaagaacttttcacaggggttgttccaatacttgttgaacttgatggggatgttaatgggcataaattctcagtttcaggagaaggagaaggagaagcgacatatgggaaacttacacttaaattcatatgtacaacagggaaacttccagttccatggccaacacttgttacaacacttacatatggagttcaatgtttctcaagatatccagatcatatgaaacaacatgatttcttcaaatcagcaatgccagaaggatatgttcaagaaagaacaatattcttcaaagatgatgggaattataaaacaagagctgaagttaaatttgaaggggatacacttgttaatagaattgaacttaaaggaattgaatttaaagaagatgggaatatacttgggcataaacttgaatataattataattcacataatgtttatattatggcagataaacaaaaaaatgggattaaagttaattttaaaattagacataatattgaagatgggtcagttcaacttgcagatcattatcaacaaaatacaccaattggggatgggccagttcttcttccagataatcattatctttcaacacaatcagcgctttcaaaagaaccaaatgaaaaaagagatcatatggttcttcttgaatttgttacagcagcagggattacacatgggatggatgaactttacaaaGGAAGCGGAGCTACTAACTTCAGCCTGCTGAAGCAGGCTGGAGACGTGGAGGAGAACCCTGG*ACCTCTCGAGT*CTCCTGCAGGcacc**ATG**CATGTACATCGACATCAACATGCTCATGCGCATGTCCATACCCATCTACATGCCCATAAACATAGGCATAGTCATCTTCATATCCATTTCCATACGCATGGACATCCTCATCGCCATTGGCATCGTCATGTTCATCAGCATTGCCATGAGCATCCGCATGGCCATTCTCATTTACATCACCATGGGCATACTCATCCCCATCATCATGTGCATATTCATCTCCATAGACATAACCATTTTCATTCCCATTTGCATGCACATAAGCATATACATGACCATCTGCATACACATAATCATGATCATAGCCATCCACATTACCATTGTCATCGGCATTATCATGGTCATTCACATGAACATTCGCAT**TAG**AGATCGGAAGAGCACACTCTAGATACCCATACGATGTACCAGATTACGCATGAGATCGGAAGAGCACACGTCTGAACTCCAGTCAC
