# Supplementary material for: Translation affects mRNA stability in a codon-dependent manner in human cells
Source: eLife. 2019 Apr 23;8:e45396. doi: 10.7554/eLife.45396 (PMC6529216; doi:10.7554/eLife.45396)
Supplement: Figure 4—source data 1. [file elife-45396-fig4-data1.docx]

**Reporter sequences and oligos used for Figure 4**

Histone tail sequence

Highlight final histone tail after processing

TGAAACTGGCGGGACGTTCCCCTTTGAAAATTTTAAACGGCTCTTTTCAGAGCCACCCACAGGTCTCAGTCAAAAGAGCTGAAGCTTTTTGGAGGGGGGAGTGGGGTGGAGAGGGGTGCGGGTGCTGCGGTGTTGTGCGGCCACGGTCTATCCTAGTCGTGCTGGTTGGGGGTCAGTTATTAACAGCTCCCAGCAGCCTGGGCGCAAGGATCCTTCCGTCTCAGTCTCAGCCACAGTCACCGGATAGCTAAGGCTACTTGAGCCACCAGGTCCTACTTTTTCTTCAAATTCCCGCGGTGGTGTGTGATTGGCCGGCCGATTTCTGTTAAAGCGCGGCGCTTAAAAGTGGCCCTGAAAAAATTTTATTTCTGACACAGGGATGTGGTGTAAATGCTTTAGTTTACTTTAGCGTTGAGGTGACTTCCTGAGCACATCCCGTCCTGGTTGTGAGCAGTACGTGGCATGCCCAAGGTGCTTCCGGCCCACGATCTTGTGCAGCTGGTCGTTGAGAATGGCCAACTGCTGGTGGTGCCCGGTGTCTGTCTTTTCGCAGGCCTTTGATAAGCCTTAGCTTTGGGATATCGGCCCTCAGGTACTGTATTTGTAAATAGTGTGGGGGCTGCACTGCGTGACGAGGTGGCTCTCCTGTAATCCCAGCATTTTGGGAGGCCGAGGCGGGGGCATCACGAGGTCAGGAGTTCGAGGCCAGCCTGAAGAACATGGTGAAGCCCCATCTCTGCTAAAAAAAAATACAAAAATTAGTCAGGCGTGGTGCCGCGCGCCTGTAATTCCAGCTACTTGGGGGCTGAGGTAGAAAATCACTTGAACCCGGGAGGCGGAAGTTGTAAGCCGAGATCGCGCCACTTGCACTCCGGCCTGGGCGATAGAACGAGACTCCGTCTCAAAATAAAAAGTGTGCCCATTGTCTTTTTCTGTCAGTGGCCAGCGTCTCCAGGACTTATTTTCCCAGGGGCTC

**Probes for Northern** Figure 4D

P2A anti probe

CACGTCTCCAGCCTGCTTCAGCAGGCTGAAGTTAG + 3’ biotin

299-GFP probe

gttctttcttgaacatatccttctggcattgctg+ 3’ biotin
