# Supplementary material for: Translation affects mRNA stability in a codon-dependent manner in human cells
Source: eLife. 2019 Apr 23;8:e45396. doi: 10.7554/eLife.45396 (PMC6529216; doi:10.7554/eLife.45396)
Supplement: Figure 5—source data 1. [file elife-45396-fig5-data1.docx]

**Reporter sequences and oligos used for Figure 5**

**5’UTR used**

5’UTR strong1

AGGGAAGCGGAGTCTGCTGTTAGTCACCGCGTGACCGCC

5’UTR strong2

GCTTGTTGGTCTTCGACAGCATTCATTTGAGCC

Middle

TCTAACGGCGAA

Weak1

TCACTGTTAAACGAATCCAAAAACATCAGGCAAA

Weak2

ATTTGTAGTTCAGCTCAGCAGCACTGACACATGGATTTACCAACTTCGTTGTTCGCTTAGATGAGCCATAAACATAAAC

**3’UTR used**

3’UTR strong

AGACCTGTTTAGATGCCATTCCAGACCTTTATTGCGGGGTGGGGGTACGGGGGGTTTGGGGAGAGGTTCGTGTCTGCATACCTTTGTTGCATGAGTGTCCTGCATATACTGTCTGAGCGGAGCCGTTTTTCCCATACATGTTCAATCTACTGTGAGGATGATGATCTGAGCTGCAGCATTGAAAACCTTCAGTCCTGTAAGGAGTTTCCAAAACCCGGCGACAGATCCGCTTTAAAGGATGTTTTGCTCTAGTATGATGGGGAAATTGGGCCAGATGTTATTTTTGTTACTTTTTAATTTCCTAACTGTTCAATCCGGCGTTTAGTGTGTGTGTGTGATGGATTTGTGAAATGCTTATGATTTTGTAAAAATCAATCAACACAAACTGTTTTACTGGTTTCTTTTGTTTGTTTTTTTCCCCTTTTGCAAAAAATATTAAACACCCTTTTGATCTT

3’UTR middle

GATCGGAAGAGCACACGTCTGAACTCCAGTCACA

3’UTR weak

GGCTCTGACACTTACAAACACTACACTTTATACACTACAACACACGCAAAGGTACACTAACGTTACGAGCGTTGCATTAAGATAGTGATTCTTTTGTTTTGTCAATATTCATTTTTATTACGTTATTAATTGTAACTCTTATTTGTAAATGTATGTATGAGTGTCTTAAGTATGTGTGTGTGTGTGTGTGTGTGACAGAGGACGATAGCACTGAAGTATTTGTTCATTTCCTTTTGGTCTTCCTGACGGGACATGTTAAAGTTTTAGCAATGCTGCAT

**5’UTR with our without uORF**

No uORF

ACAGCACGATCAGGGATCTTCAGCTTTAACTGTTCAAGACACTCGATCAGGGAAGTCTGTACATAGTCCAAAGTCGATCAGGGGTATTGCTCTTTAGGCCACCATGGTGAGTAA

1uORF weak

ACAGCACGATCAGGGATCTTCAGCTTTAACTGTTCAAGACACTCGtTtAtGGAAGTCTGTACATAGTCCAAAGTCGATCAGGGGTATTGCTCTTTAGGCCACCATGGTGAGTAA

3uORF

ACAGCACGATCAtGGATCTTCAGCTTTAACTGTTCAAGACACTCGATCAtGGAAGTCTGTACATAGTCCAAAGTCGATCAtGGGTATTGCTCTTTAGGCCACCATGGTGAGTAA

1uORF

ACAGCACGATCAGGGATCTTCAGCTTTAACTGTTCAAGACACTCGATCAtGGAAGTCTGTACATAGTCCAAAGTCGATCAGGGGTATTGCTCTTTAGGCCACCATGGTGAGTAA

1oORF

ACAGCACGATCAGGGATCTTCAGCTTTAACTGTTCAAGACACTCGATCAtGGAAGTCTGTACATtGTCCAAAGTCGATCAGGGGTATTGCTCTTTAGGCCACCATGGTGAGTAA

**Primers for qPCR**

mChery 5’

GGATGGGAAGCGTCATCAGA

mCherry 3’

AGCATCGTAATGCCCTCCAT

GFP 5’

TGGGTCAGTTCAACTTGCAG

GFP 3’

GAAAGCGCTGATTGTGTTGA
